# Structural determination and modeling of ciliary microtubules

**DOI:** 10.1107/S2059798324001815

**Published:** 2024-03-07

**Authors:** Travis Walton, Matthew H. Doran, Alan Brown

**Affiliations:** a Department of Biological Chemistry and Molecular Pharmacology, Blavatnik Institute, Harvard Medical School, Boston, MA 02115, USA; University of Leeds, United Kingdom

**Keywords:** cryo-EM, cryo-ET, cilia, axonemes, microtubules

## Abstract

The axoneme, a microtubule-based array at the center of every cilium, has been the subject of structural investigations for decades, but only recent advances in cryo-EM and cryo-ET have allowed a molecular-level interpretation of the entire complex to be achieved. The unique properties of the nine doublet microtubules and central pair of singlet microtubules that form the axoneme, including the highly decorated tubulin lattice and the docking of massive axonemal complexes, provide opportunities and challenges for sample preparation, 3D reconstruction and atomic modeling. Here, the approaches used for cryo-EM and cryo-ET of axonemes are reviewed, while highlighting the unique opportunities provided by the latest generation of AI-guided tools that are transforming structural biology.

## Abstract 2

This review describes how electron-microscopy methods have helped to reveal the structure of the ciliary axoneme.

## 1. Introduction

### 1.1. The structure of the axoneme comes into view

**Figure 1:**
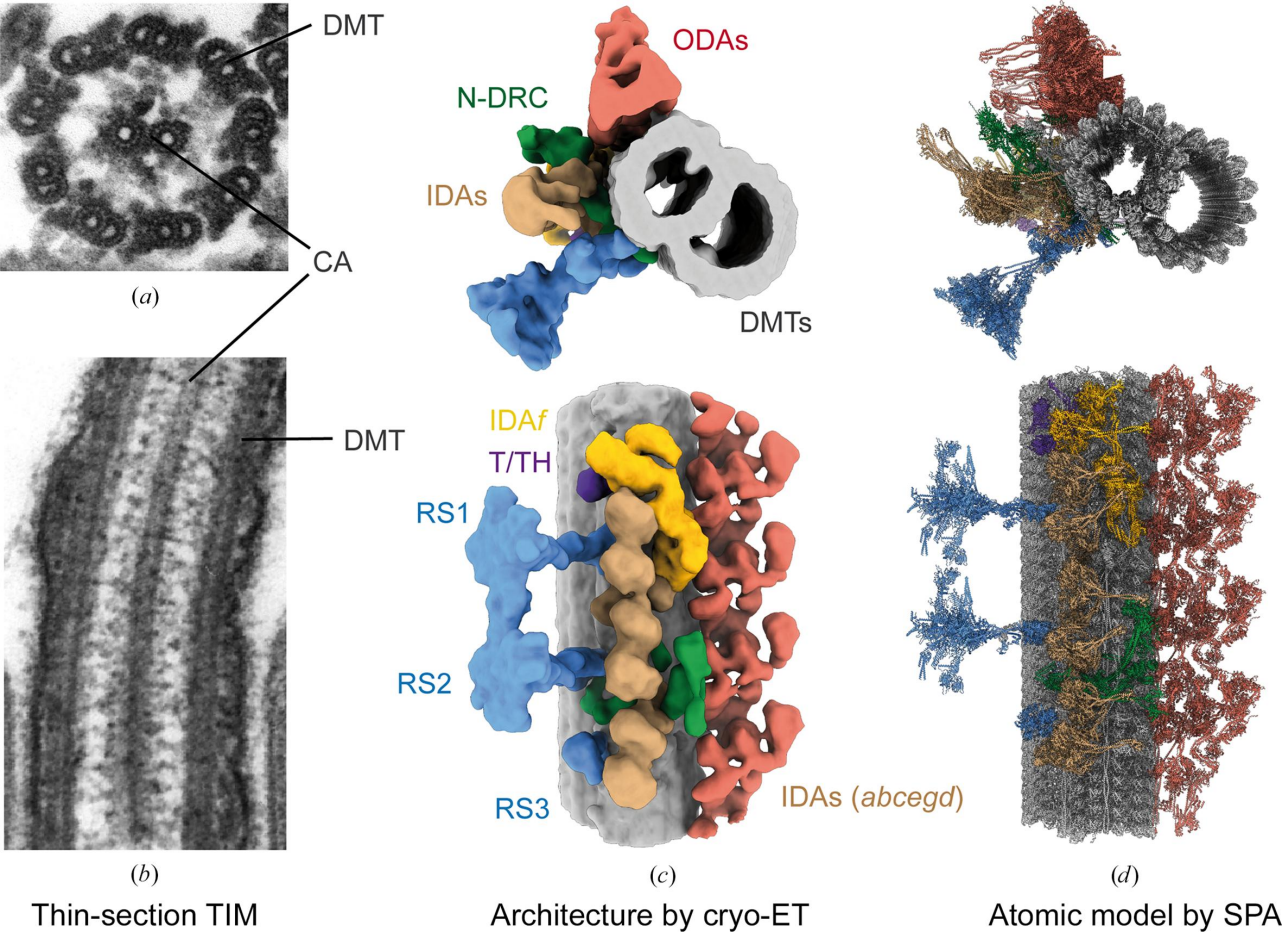
The use of electron microscopy to visualize ciliary axonemes. (*a*) Resin-embedded, thin-section transmission electron microscopy (TEM) revealed the 9 + 2 architecture of many axonemes, in which nine doublet microtubules (DMT) encircle a central apparatus (CA) of two singlet microtubules. (*b*) Thin-section TEM also revealed periodic projections from both the CA and DMT. These images were obtained from https://commons.wikimedia.org. (*c*) Cryo-ET and subtomogram averaging of axonemes revealed the architecture of the 96 nm repeat unit of the DMT, providing the location and shape of many axonemal complexes including outer dynein arms (ODAs), inner dynein arms (IDAs), radial spokes (RS), the nexin–dynein regulatory complex (N-DRC) and the tether/tetherhead (T/TH) complex. EMDB entry EMD-2115 is shown colored by complex (Bui *et al.*, 2012 ▸). (*d*) Single-particle analysis cryo-EM of splayed axonemes from *C. reinhardtii* allowed an atomic model of the 96 nm repeat to be built (PDB entry 8glv; Walton *et al.*, 2023 ▸)*.*

Our understanding of axonemal structure is intertwined with developments in electron microscopy. Early transmission electron-microscopy (TEM) studies, using thin-sectioned, resin-embedded samples, described the canonical 9 + 2 microtubule architecture of the axoneme of motile cilia, with nine doublet microtubules (DMTs) encircling a central apparatus (CA) of two singlet microtubules (Taira & Shibasaki, 1978 ▸; Afzelius, 1955 ▸; Fawcett & Porter, 1954 ▸; Fig. 1 ▸ *a*). Similar experiments on nonmotile (primary) cilia revealed a 9 + 0 architecture comprising DMTs without the CA (Barnes, 1961 ▸). These and further studies provided the first views of the DMT, revealing an A tubule with 13 protofilaments connected to a B tubule with ten protofilaments. For motile cilia, they also revealed periodic complexes projecting from the microtubule surfaces (Fig. 1 ▸ *b*). Each axonemal DMT was shown to be composed of 96 nm repeating units that feature two rows of dynein motors – outer and inner dynein arms – that power the ciliary beat. Additional regulatory complexes such as radial spokes (RSs) and the nexin–dynein regulatory complex (N-DRC) form connections with adjacent dyneins to adjust the waveform dynamics in response to mechanical cues (Witman *et al.*, 1978 ▸; Bozkurt & Woolley, 1993 ▸).

Later work eliminated the need for chemical fixation by imaging cryopreserved cilia and ciliated cells (Nicastro *et al.*, 2005 ▸, 2006 ▸; Sui & Downing, 2006 ▸). Because of the size and the intricate architecture of the axoneme, the first cryoelectron microscopy (cryo-EM) studies of *Chlamydomonas reinhardtii* and sea urchin sperm axonemes were studied by tomography (cryo-ET), in which the samples were imaged from multiple angles to create a tilt series that could be aligned and interpolated to create a three-dimensional tomogram. Subtomogram averaging (STA) exploited the periodicity of the DMT to enhance the resolution, improve signal-to-noise ratios and mitigate anisotropy. These refined averages were the first to distinguish the orientation of the dyneins and their connections to other axonemal complexes (Nicastro *et al.*, 2005 ▸, 2006 ▸; Bui *et al.*, 2012 ▸; Fig. 1 ▸ *c*), providing valuable information about the regulatory mechanisms that coordinate dynein activity. In addition to these large features, tomographic reconstructions also identified densities periodically associated with the inside walls of the DMT now known as ‘microtubule inner proteins’ or ‘MIPs’ that repeat with a periodicity of up to 48 nm (Sui & Downing, 2006 ▸; Nicastro *et al.*, 2006 ▸). These were initially speculated to reinforce the protofilament network and dock axonemal complexes, a prescient suggestion that was later substantiated by functional and structural studies (Nicastro *et al.*, 2006 ▸; Owa *et al.*, 2019 ▸; Ichikawa *et al.*, 2017 ▸, 2019 ▸).

These pioneering cryo-ET studies significantly improved our understanding of axonemal ultrastructure. However, it was only upon the introduction of cryo-EM with single-particle analysis (SPA) that subnanometre reconstructions became possible. SPA removed the need to tilt the sample during data acquisition, which led to higher throughput, less electron damage, improved signal-to-noise ratios and, ultimately, higher resolution. Applied to the DMT, advances in high-resolution imaging, sample preparation and particle-alignment workflows culminated in the reconstruction of the 48 nm repeat of the *C. reinhardtii* DMT at ∼3.5 Å resolution (Ma *et al.*, 2019 ▸) and the generation of an atomic model (Fig. 1 ▸ *d*). Once this was achieved, the application of SPA cryo-EM to DMT complexes from various species and cell types resulted in a boon of reconstructions that have proven to be key in establishing the identities, positions, repeat lengths, structures and interactions of DMT-associated proteins (Gui, Farley *et al.*, 2021 ▸; Walton *et al.*, 2023 ▸; Kubo *et al.*, 2023 ▸; Leung *et al.*, 2023 ▸; Zhou *et al.*, 2023 ▸; Gui, Croft *et al.*, 2022 ▸). Information from these structures has provided invaluable insights into the function of axonemal proteins, the evolution of the axoneme and the molecular underpinnings of ciliopathies.

### 1.2. Reconstruction of axonemal microtubules

There are several hurdles that make determination of the structure of axonemal microtubules by SPA cryo-EM difficult. Imaging the axoneme directly produces projection images with overlapping DMT and CA microtubules, complicating particle picking and alignment (Cheng *et al.*, 2015 ▸). Methods for deconstructing the axoneme into its constitutive filaments have proven to be successful in separating axonemal microtubules, but typically result in the degradation or loss of native interactions. To overcome the challenge of sample preservation, axonemes can be gently splayed into constitutive filaments suitable for cryo-EM imaging while retaining the majority of axonemal proteins (Walton *et al.*, 2021 ▸, 2023 ▸).

In addition to the difficulties introduced by sample preparation for cryo-EM imaging, particle alignment of DMTs is not straightforward, mainly due to their 96 nm repeat units. During particle picking, the register of these units along the microtubule filament is unclear, so any given point along a DMT filament will be at an indeterminate position within the 96 nm repeat. Therefore, to accurately align particles with the correct periodicity, the local register of the particles must be identified through a series of 3D classifications starting from 8 nm picked particles. As a result, particles can be aligned with periodicities of 24, 48 or 96 nm, which are sufficient to resolve the majority of DMT-associated proteins.

The last challenge in determining the structure of DMTs is the construction of atomic models. This process involves identifying axonemal proteins, generating initial models, integrating them within the larger complex and refining them against the cryo-EM density. Because the axoneme is a complex comprising several hundred different proteins, many of whose structures have not been determined, each step in this process is demanding. However, the challenge of model building has waned with improvements in automated, AI-driven model-building methods.

This review will summarize the advances that made atomic models of axonemal microtubules possible. We will describe sample preparation for SPA imaging, review the image-processing procedure and discuss methods for protein identification and model building for cryo-EM density. Finally, we will evaluate the exciting new developments that make cryo-ET poised to re-emerge as the premier method for axonemal structure determination.

## 2. Cryo-EM single-particle analysis of cilia components

Drawing upon decades of genetic, biochemical and structural studies, SPA cryo-EM of the axoneme has recently achieved a significant milestone: a near-complete atomic model of the axoneme for the model organism *C. reinhardtii* (Walton *et al.*, 2023 ▸; Fig. 1 ▸ *d*). This journey began with cryo-EM studies characterizing the α- and β-tubulin lattice of the DMT (Ichikawa *et al.*, 2017 ▸; Maheshwari *et al.*, 2015 ▸), and soon thereafter reached resolutions enabling the identification of MIPs in DMTs from both *C. reinhardtii* and *Tetrahymena thermophila* (Ma *et al.*, 2019 ▸; Khalifa *et al.*, 2020 ▸). The determination of the 48 nm DMT repeat structure in *C. reinhardtii* revealed a network of interactions that establish periodic patterns of MIP binding to the tubulin lattice, providing insight into its ability to withstand mechanical stresses during ciliary beating (Ma *et al.*, 2019 ▸). Subsequently, structures of the 48 nm DMT repeat have been determined for human and bovine respiratory cilia (Gui, Farley *et al.*, 2021 ▸; Gui, Ma *et al.*, 2021 ▸), and the sperm cilia of sea urchin (Leung *et al.*, 2023 ▸) and various mammals (Leung *et al.*, 2023 ▸; Zhou *et al.*, 2023 ▸), providing insight into the evolutionary specialization of DMT structure.

Structures of native axonemal complexes (including dynein motors, radial spokes and the nexin–dynein regulatory complex) followed shortly thereafter, using strategies that either imaged complexes directly on DMTs (Gui, Farley *et al.*, 2021 ▸; Walton *et al.*, 2023 ▸; Kubo *et al.*, 2023 ▸; Ghanaeian *et al.*, 2023 ▸) or following biochemical dissociation from the axoneme (Gui, Ma *et al.*, 2021 ▸; Rao *et al.*, 2021 ▸). These reconstructions were complemented by structures of radial spoke heads prepared through *in vitro* reconstitution (Grossman-Haham *et al.*, 2021 ▸; Zheng *et al.*, 2021 ▸). Together with structures of the CA from *C. reinhardtii* (Gui, Wang *et al.*, 2022 ▸; Han *et al.*, 2022 ▸), our comprehension of the axoneme at the molecular level has undergone a profound transformation in the past six years.

### 2.1. Sample preparation for SPA imaging

Early studies of microtubule structure by cryo-EM relied upon *in vitro* reconstitution (Nogales, 2015 ▸); however, this approach is unsuitable for DMTs or the CA due to the large number of microtubule-associated factors and their complex patterns of localization. Instead, studies of axonemal structure have traditionally used native isolation techniques, leveraging the exceptional stability of ciliary microtubules. Motile cilia can be removed from cells by a combination of dibucaine treatment, millimolar levels of Ca^2+^ or mechanical force, and then crudely separated from cell debris by centrifugation over sucrose cushions (Craige *et al.*, 2013 ▸; Beneke *et al.*, 2020 ▸). Mild detergent treatment followed by pelleting yields bare axonemes, which must be further broken apart before imaging by SPA to avoid densely overlapping microtubules. The earliest studies of the *Tetrahymena* DMT by cryo-EM broke the axoneme apart using a combination of ATP and NaCl washes (Ichikawa *et al.*, 2017 ▸; Maheshwari *et al.*, 2015 ▸). ATP activates the axonemal dyneins to help slide apart the microtubules of the axoneme, while the high-salt washes remove the axonemal dyneins and break the electrostatic interactions that hold neighboring DMTs together. In addition, radial spokes were removed using dialysis of the pelleted DMTs after salt extraction and the DMTs were broken into fragments by sonication.

An alternative to NaCl is to use mild protease digestion to sever the linkages between DMTs, followed by the addition of ATP to telescope apart the axoneme through DMT sliding. This process, called sliding disintegration, has historical precedent in the seminal work of Summers & Gibbons (1971 ▸), which corroborated observations by Satir that DMTs move relative to each other during ciliary beating (Satir, 1967 ▸). Throughout the decades, a variety of proteases, including subtilisin A, which cleaves the tubulin C-terminal tail (Sackett *et al.*, 1985 ▸), and reaction conditions have been used to digest the axoneme for biochemical and structural studies. To minimize the damage caused by digestion, sliding disintegration is often performed on ice in the presence of protease inhibitors. This approach yielded the first sub-4 Å resolution structure of the DMT 48 nm repeat (Ma *et al.*, 2019 ▸) and, soon after, the radial spoke and CA complexes (Gui, Ma *et al.*, 2021 ▸; Gui, Wang *et al.*, 2022 ▸).

#### 2.1.1. Axoneme splaying to retain tubulin decoration

A major limitation of cryo-EM sample preparation by salt extraction or sliding disintegration is the loss of, or damage to, axonemal complexes, which hindered structural determination of the docking and regulatory interactions that are important for ciliary beating. An alternative approach with the potential to retain more native interactions was suggested by studies of *in vitro* reactivation of ciliary beating in detergent-extracted cell models and isolated axonemes (Kamiya & Okagaki, 1986 ▸; Aoyama & Kamiya, 2005 ▸; Mukundan *et al.*, 2014 ▸). Prolonged reactivation with exogenous ATP resulted in fraying of the axoneme distal end, and sometimes extensive splaying along the length of the axoneme. This splaying was exacerbated by mutations in the N-DRC, which links neighboring DMTs together through electrostatic interactions involving tubulin glutamylation (Bower *et al.*, 2013 ▸; Alford *et al.*, 2016 ▸). Optimization of *in vitro* reactivation conditions, such as the ATP, calcium and axoneme concentrations, for cryo-EM of wild-type axonemes led to structures of outer dynein arms (ODAs) natively docked onto DMTs (Walton *et al.*, 2021 ▸; Kubo *et al.*, 2021 ▸), and eventually the near-total complement of axonemal complexes in the 96 nm repeat network (Walton *et al.*, 2023 ▸). These findings indicate that axoneme splaying largely preserves the 96 nm repeat, making it a valuable method to study DMT structure.

#### 2.1.2. Central apparatus extrusion

An elegant approach to preparing the CA for cryo-EM analysis entails its enrichment through ATP-induced extrusion from isolated axonemes without protease treatment. Early work demonstrated that ATP-induced reactivation of isolated axonemes first causes projection and rotation of the CA and that this process can be driven to completely eject the CA from the axoneme (Kamiya, 1982 ▸). A recent cryo-EM study of the CA improved the extrusion protocol by using multiple additions of ATP and prolonged incubation to enhance the yield (Han *et al.*, 2022 ▸). Low-speed centrifugation removed bundles of DMTs, and the supernatant was then centrifuged at higher speeds to concentrate the CA microtubules for cryo-EM grids. Note that this approach may only work for species that have CAs that are capable of rotating.

### 2.2. Cryo-EM image processing

**Figure 2:**
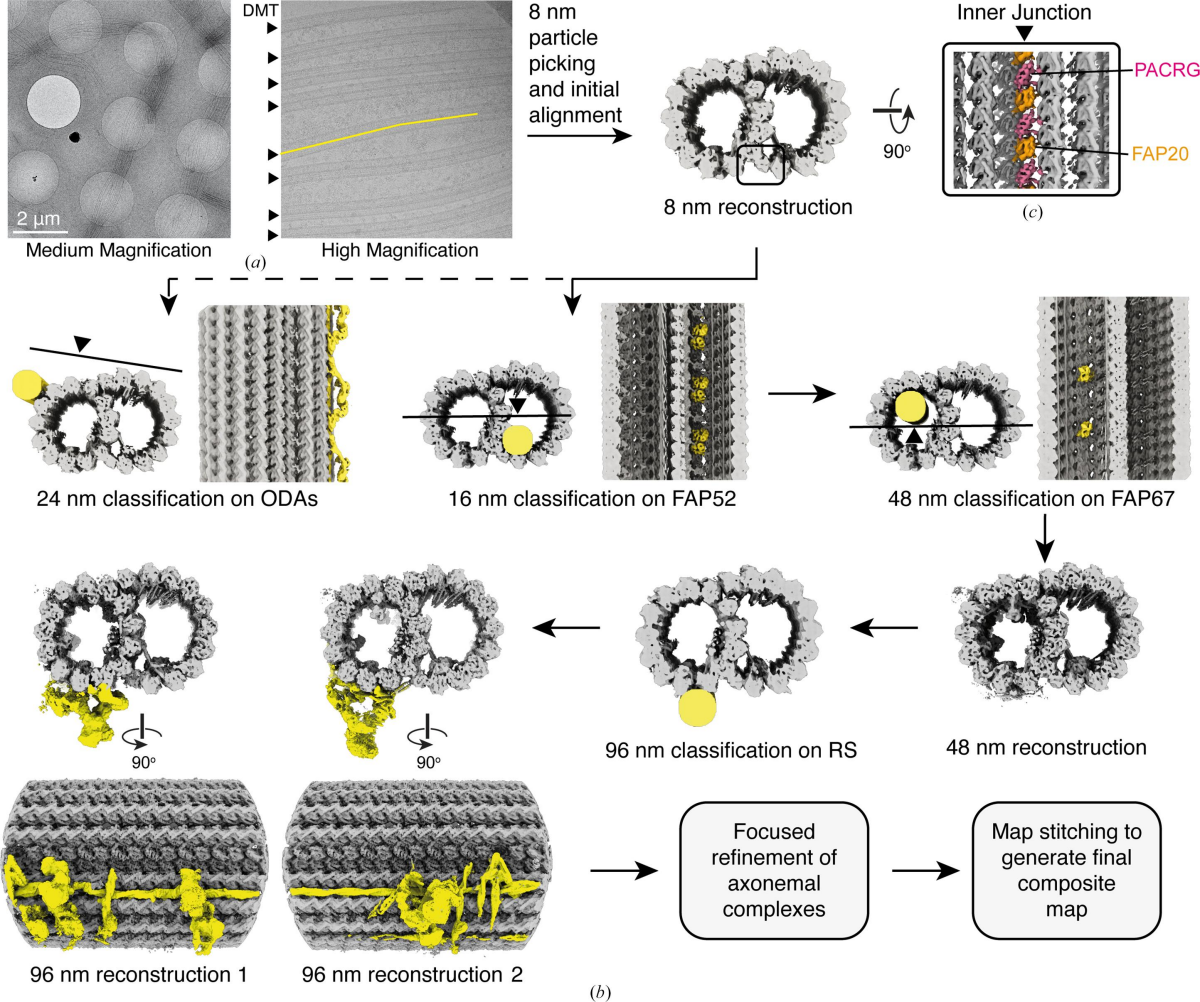
The cryo-EM processing scheme used to reconstruct doublet microtubules (DMTs). (*a*) Electron micrographs showing splayed axonemes at medium magnification (left) and DMTs (each marked with an arrowhead) at high magnification (right). The DMTs are picked (illustrated with a yellow line for a single DMT) prior to particle extraction. (*b*) Schematic showing the steps taken to reconstruct DMTs at progressively higher periodicities, starting with particles extracted every 8 nm along the longitudinal axis of the microtubule. Each step relies on mask-focused classification with masks (illustrated as yellow cylinders) covering features known to have a higher periodicity than the map to which they are applied. For example, 16 nm periodicity can be obtained by classifying on the position of FAP52 (a microtubule inner protein with 16 nm periodicity) in the 8 nm reconstruction. (*c*) The quality of the initial alignment can be monitored by inspecting the inner junction: a repeating pattern of FAP20 and PACRG. These proteins should be clearly distinguishable from one another in well aligned 8 nm reconstructions.

To reconstruct the DMT, a specialized image-processing pipeline is required due to the periodic nature of axonemal complexes bound to the tubulin surface. This processing differs from the microtubule-reconstruction methods developed for cytosolic microtubules (Zhang & Nogales, 2015 ▸; Cook *et al.*, 2020 ▸), which are primarily concerned with determining the number of protofilaments (as cytosolic and especially *in vitro* reconstituted microtubules can have variable numbers of protofilaments) and the location of the microtubule seam (a break in the helical symmetry of the filament where heterotypic α–β and β–α lateral contacts occur) (Chrétien & Wade, 1991 ▸; Kikkawa *et al.*, 1994 ▸; Ray *et al.*, 1993 ▸; Kellogg *et al.*, 2017 ▸). Neither of these concerns are particularly relevant to DMTs, which have a set number of protofilaments distributed between A and B tubules, and large projections that create features that preclude the need to specifically locate the seam. In this section, we will outline the image-processing workflow used to reconstruct the axonemal DMT (Fig. 2 ▸).

#### 2.2.1. Data collection

The main challenge for most SPA data collections is identifying regions of the grid that have suitably thin ice and contain enough particles for imaging. A similar process must be performed for samples containing axonemal DMTs and CAs. Typically, the concentrations of these large filaments are relatively low to avoid crossovers and bundling, which means that some grid holes will be empty. However, because DMTs and CAs are large, with individual DMTs measuring 30 nm in width and several micrometres in length, each filament can be seen at medium magnification (Fig. 2 ▸), allowing users to target the sample by selecting holes that contain DMTs or manually deselecting holes without sample. Despite being time-consuming, this process ensures that most images contain samples suitable for image processing.

#### 2.2.2. Particle picking

Particle picking is a vital step in any cryo-EM image-processing workflow, crucially selecting the particle projections to be aligned later. It is important to select particles of interest while minimizing the inclusion of false positives. Picking filaments is particularly challenging due to the need to avoid crossed or overlapping filaments. Although manual selection of the start and end points of filaments remains perhaps the most accurate method of particle picking for filaments, high-throughput data sets of tens of thousands of images renders this time-intensive method impractical in many cases. Because of this, numerous automatic particle-picking software have arisen in recent years that work at human levels of accuracy, but at a much faster pace. Both template/blob pickers (for example the *cryoSPARC* filament tracer) and deep-learning-based particle-picking programs (for example *crYOLO*) have successfully been used to pick DMT particles (Leung *et al.*, 2023 ▸; Zhou *et al.*, 2023 ▸; Fig. 2 ▸ *a*).

In addition to ‘off-the-shelf’ automated particle-picking algorithms, other pipelines have been made specifically for microtubule-bound complexes. For example, the Zhang laboratory has developed a pipeline for the accurate particle picking of microtubules bound by proteins in a process called ‘multi-curve’ fitting (Chai *et al.*, 2022 ▸). In this workflow, ‘seeding particles’ are initially picked using conventional methods. Once a seeding particle has successfully been found, filaments are traced by absorbing the nearest individual particles. Distances between particles and fitting errors between them are used as criteria to accept the growing filaments, resulting in the identification of microtubule filaments despite their curvature or associated proteins. The development of multiple automated approaches means that the picking of DMT filaments is now no longer a bottleneck in the cryo-EM processing pipeline.

#### 2.2.3. Determination of 48 and 96 nm repeat structures

After selecting filaments from the micrographs, they are cut into 8 nm overlapping segments during particle extraction to match the periodicity of the α/β-tubulin heterodimer (Fig. 2 ▸ *b*). Initial alignments of these particles, in which each 8 nm segment is treated as a conventional single particle, can be performed in *FREALIGN* (Grigorieff, 2016 ▸), *RELION* (Zivanov *et al.*, 2018 ▸, 2022 ▸) or *cryoSPARC* (Punjani *et al.*, 2017 ▸), and should resolve the A and B tubules of the DMT with a periodicity of 8 nm. The quality of the initial alignment can be gauged by inspecting the inner junction, a repetitive pattern of FAP20 and PACRG linking the A and B tubules (Fig. 2 ▸ *c*). Subsequent classification on the inner junction can identify misaligned or damaged particles for removal at this step. In practice, this 8 nm reconstruction lacks clear density for most of the associated MIPs and axonemal complexes, which have higher order periodicities. The lack of these features is caused by averaging particles from 12 different registers of the 96 nm repeat. The challenge of the DMT image-processing workflow is to resolve the higher periodicity features by progressively identifying the periodicity of each particle.

Existing knowledge of the DMT structure has been key to achieving high-order periodic reconstructions. Tomographic studies have shown that internal MIPs have specific periodicities, such as 16 and 48 nm, whereas external axonemal complexes have periodicities of 24 and 96 nm (Nicastro *et al.*, 2006 ▸, 2011 ▸; Sui & Downing, 2006 ▸). Given that the locations of these densities within the DMT are known, targeted classifications at these markers can be used to determine the periodicity of particles. To make this process manageable, we have observed that identifying and obtaining reconstructions at progressively higher periodicities of up to 96 nm is the best practice (Fig. 2 ▸ *b*). For example, from initial 8 nm reconstructions, we can classify on the well known 16 nm MIP FAP52, which is situated in the B tubule adjacent to the inner junction. The resulting classification should yield two well resolved classes showing FAP52 and other 16 nm MIP densities, offset by 8 nm. Once these classes have been resolved, they can be combined by shifting one set of the particles by 8 nm. There are multiple methods for shifting particles, but utilizing the re-extraction and re-centering feature in *RELION* offers a convenient approach. Post-merging, it is important to remove any duplicated particles to prevent overinflated resolution estimations. Subsequent refinement of the merged particle set will result in a 16 nm reconstruction with conspicuous density for 16 nm periodic components and diffuse density for higher periodic components. This process of classification on known components followed by particle merging is the basis for reconstructing higher order periodic components of the DMT, eventually leading to the 96 nm repeat. From the 16 nm reconstruction, the 48 nm repeat can be obtained by classifying on FAP67, an A-tubule MIP which in *C. reinhardtii* has two copies within a 48 nm repeat (Ma *et al.*, 2019 ▸). From the 48 nm reconstruction, the 96 nm repeat can be obtained by focusing classification on the external radial spoke complexes, which exhibit periodicities of 96 nm. For certain components such as the ODAs, with a 24 nm repeat (Walton *et al.*, 2021 ▸), achieving their specific periodicity may be sufficient to produce adequate maps for model building without needing to resolve higher periodicities (Fig. 2 ▸ *b*).

An important caveat to these classifications is that due to poor initial 8 nm alignment of DMT samples, many particles can be unnecessarily discarded. This can reduce the number of particles included in the final higher periodicity reconstructions, limiting the resolution and map quality. To mitigate this issue, a shifting strategy can be implemented to retain more particles. In this scheme, the coordinates of the final particles are used to locate adjacent positions on the same DMT filaments (Walton *et al.*, 2021 ▸, 2023 ▸). By inheriting alignment parameters from neighboring ‘well behaved’ particles, these particles can be added to the data set, with duplicates deleted, and contribute to final reconstructions to produce higher quality maps.

#### 2.2.4. Focused refinements

Due to the considerable size of the 96 nm repeat of the DMT, computational constraints preclude the high-resolution reconstruction of the full complex. In addition, local flexibility, differences in the occupancy of axonemal components and local misalignments produce resolution variations across the DMT reconstruction. To ameliorate these issues, it is advisable to focus on individual axonemal complexes by particle subtraction, classification and refinement. By concentrating refinement on individual subregions, the map quality and resolution can improve dramatically. The full complex can then be reconstituted *in silico* by combining each sub-refinement, yielding a much-improved final map. Such schemes have been very successful in producing reconstructions that are suitable for atomic model building (Walton *et al.*, 2023 ▸; Gui, Wang *et al.*, 2022 ▸; Han *et al.*, 2022 ▸).

Sub-refinements were first applied to the 48 nm reconstruction of the *C. reinhardtii* DMT (Ma *et al.*, 2019 ▸). Here, the DMT was divided into subregions using ten cylindrical masks that each contained two or three protofilaments. Each sub­region was subsequently divided into top, middle and bottom sections using shorter cylindrical masks to create a total of 30 subregions. Each subregion was subtracted from the consensus reconstruction and refined individually. By focusing alignment on these local regions, the resolution and map quality of the tubulin and associated proteins improved substantially. The improved maps were aligned and resampled to the 48 nm consensus reconstruction and merged together by taking the maximum density value at each voxel in *ChimeraX* (Pettersen *et al.*, 2021 ▸). Alternatively, *Phenix* offers the tool combine_focused_maps that combines focused cryo-EM maps using models to align them (Liebschner *et al.*, 2019 ▸). This method was successfully used to merge focused maps from the *T. thermophila* DMT (Kubo *et al.*, 2023 ▸).

This focused refinement strategy can also be implemented for axonemal complexes bound to the exterior of the DMT or CA microtubules. Because many of these complexes project far from the microtubule surface, they are more flexible and may have lower occupancy due to damage during sample preparation and grid freezing. It follows that subtraction and subsequent focused 3D classification and refinement will improve the resolution and quality of these regions. This strategy was successful in resolving the structures of each axonemal complex with 96 nm periodicity to produce a composite reconstruction of the full repeat in both *C. reinhardtii* and human respiratory DMTs (Walton *et al.*, 2023 ▸).

In addition to improving map quality, particle classification after signal subtraction and focused refinement can reveal conformational heterogeneity. For example, extensive classification of *C. reinhardtii* IDA*f* and N-DRC revealed multiple conformation states that are coupled to movement of adjacent ODAs and which may be important for the mechanism of ODA activation (Walton *et al.*, 2023 ▸). Applying this workflow to other parts of the axoneme may uncover additional conformational states.

Signal subtraction can also be used to improve the alignments of axonemal complexes by subtracting the microtubule signal directly. In many cases, the DMT dominates alignments, smearing the alignment of bound complexes, especially if they project far from the surface of the DMT. In a method developed by the Zhang group, subtraction of the DMT density directly from micrographs after 4 nm sampling and tracing of the filaments can yield large improvements in the alignment of the bound complexes (Chai *et al.*, 2022 ▸). This method produced suitable 2D class averages and high-resolution reconstructions of ODAs reconstituted on microtubules. A similar method of tubulin subtraction was used to resolve the structure of a cytoskeletal dynein–dynactin in the presence of an adaptor bound to microtubules (Chaaban & Carter, 2022 ▸). Overall, signal subtraction, followed by focused refinement and classification, can greatly improve the map resolution and quality of axonemal complexes to allow model building.

## 3. Protein identification and model building

### 3.1. Protein identification pre-*AlphaFold*

**Figure 3:**
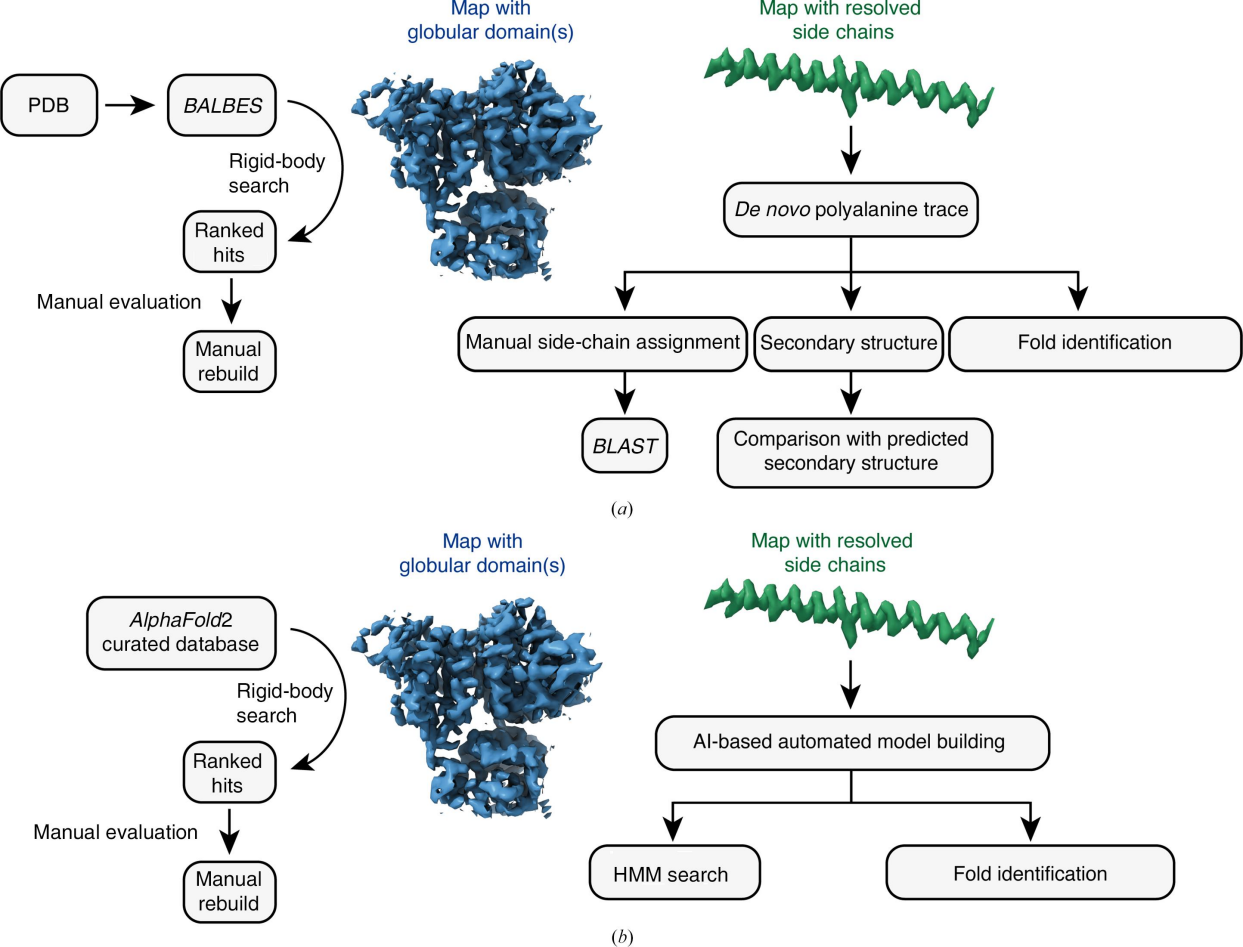
Strategies for building atomic models. (*a*) General scheme for interpreting cryo-EM maps of unknown identity before 2021. The protein fold responsible for a region of globular density could be determined by evaluating the fit of a library of protein folds (the *BALBES* database) curated from the PDB to the density (Brown *et al.*, 2015 ▸; Vagin & Teplyakov, 2010 ▸). Map regions with resolved side chains could be built manually, with side-chain predictions used to generate query sequences for *BLAST* searches against proteomes. Alternatively, secondary structure could be extracted from the built model and compared with precomputed profiles, or the tertiary structure compared with PDB entries using *DALI* (Holm, 2022 ▸) or *PDBeFold* (Krissinel & Henrick, 2004 ▸) to identify likely folds. (*b*) Current best approaches for interpreting cryo-EM maps. The protein fold responsible for a region of globular density can be determined by evaluating the fit to the density of *AlphaFold*2-created model libraries of entire proteomes (Jumper *et al.*, 2021 ▸). Map regions with resolved side chains can be built automatically using AI-based tools such as *ModelAngelo* (Jamali *et al.*, 2024 ▸) or *DeepTracer* (Pfab *et al.*, 2021 ▸; Chang *et al.*, 2022 ▸). Sequence assignments can be extracted and used to create profile hidden Markov models (HMMs) for proteome searches. Alternatively, the three-dimensional models can be compared with millions of predicted structures using *Foldseek* (van Kempen *et al.*, 2023 ▸) to identify the exact protein or protein fold.

The first ∼3.5 Å resolution structure of a DMT presented a formidable model-building challenge (Ma *et al.*, 2019 ▸). Although the tubulin component could easily be modeled by substituting the sequence from previously solved cytosolic microtubule structures and manually refitting the model to the map, the dozens of MIPs had to be identified directly from the cryo-EM density. For globular MIP densities, the *MOLREP*–*BALBES* pipeline, a density-based fold-recognition workflow (Brown *et al.*, 2015 ▸; Vagin & Teplyakov, 2010 ▸), was used (Fig. 3 ▸ *a*, left). In this workflow, MIP densities were extracted from the composite map and each entry in the *BALBES* database (Long *et al.*, 2008 ▸), a database of unique protein folds derived from the Protein Data Bank (PDB), was systematically fitted to the extracted densities using *MOLREP* (Vagin & Teplyakov, 2010 ▸). The solutions were then ranked and the top hits manually inspected to identify the best-fitting domain. Cross-referencing the recognized fold with mass-spectrometric analysis of the cryo-EM sample typically identified a MIP with a similar domain, while side-chain analysis differentiated the correct solution. This semi-automated approach was successful in identifying FAP67, FAP161, RIB72, FAP363, FAP182 and multiple EF-hand motifs (Ma *et al.*, 2019 ▸). Once the proteins have been identified in one species, homology-modeling programs such as *SWISS-MODEL* and *I-TASSER* (Schwede *et al.*, 2003 ▸; Zhang, 2008 ▸) can be used to build starting models for DMTs from other species.

Nonglobular and remaining MIP densities were built *de novo* (Fig. 3 ▸ *a*, right), starting with polyalanine traces built manually in graphical programs such as *Coot* (Emsley *et al.*, 2010 ▸). These initial traces were then used as templates to assign side chains or secondary structure: MIPs could be identified by comparing the assigned sequence against proteomes (either of the whole organism or a subset of proteins obtained from mass-spectrometric analysis of the sample) or comparing the secondary-structure profile with predictions. The traced models could also be used as an input for search programs such as *PDBeFold* (Krissinel & Henrick, 2004 ▸) and *DALI* (Holm, 2022 ▸) to identify structurally similar proteins pre-existing in the PDB. As in the example of the *MOLREP*–*BALBES* pipeline, knowledge of the protein fold can be used to identify proteins with similar domains in the mass-spectrometric results, simplifying the search space. The recent development of *Foldseek* means that this approach can now be used to search millions of predicted structures (van Kempen *et al.*, 2024 ▸).

### 3.2. Developments in protein identification and model building

**Figure 4:**
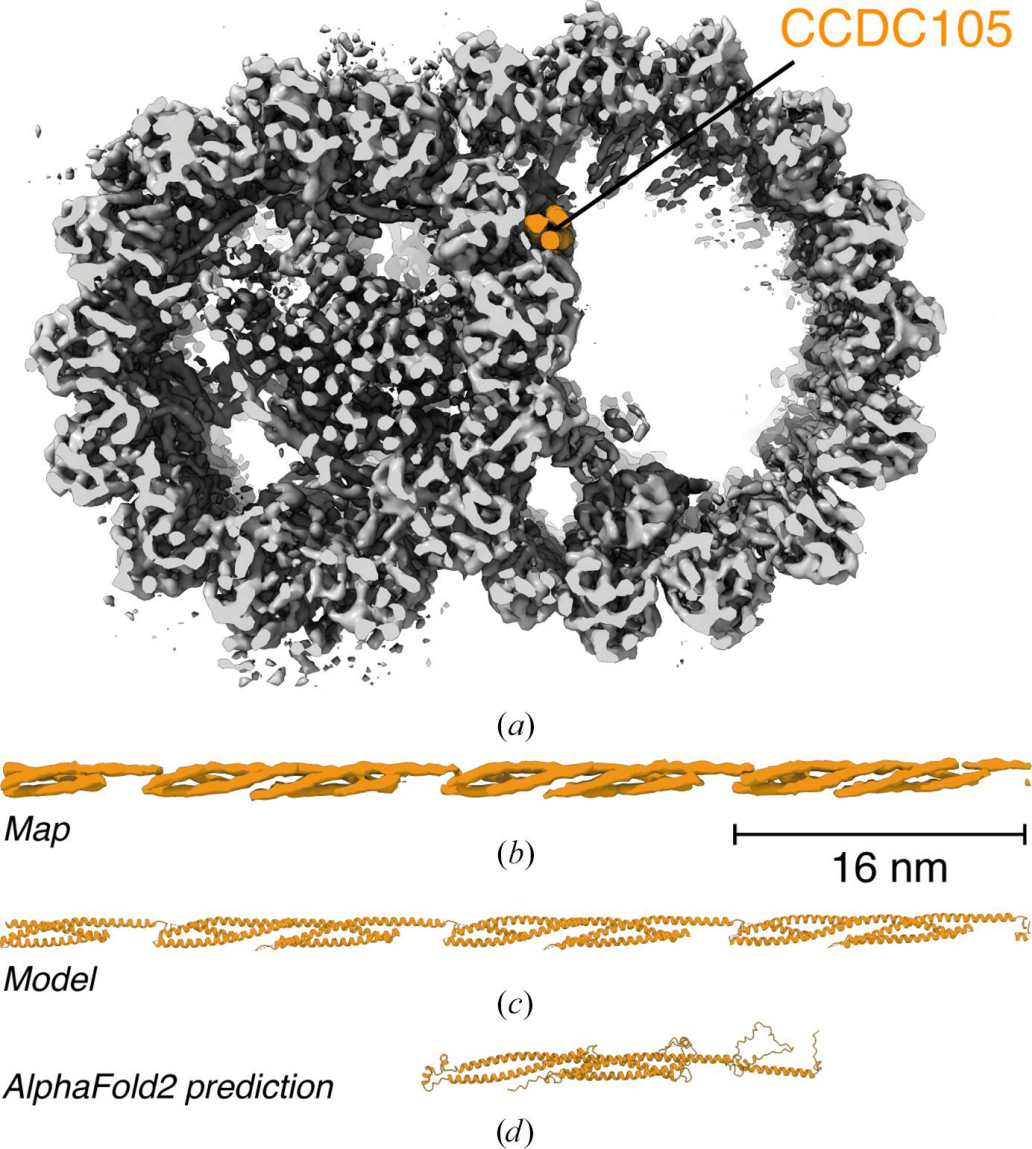
Subtomogram average of the mouse sperm doublet microtubule (DMT) and identification of CCDC105. (*a*) Cross-sectional view of the 48 nm repeat of the mouse sperm DMT determined using *in situ* cryo-ET and subtomogram averaging (EMDB entry EMD-41431; Chen, Shiozaki *et al.*, 2023 ▸). The overall resolution is estimated to be 7.7 Å. CCDC105, a microtubule inner protein of the B tubule, is shown in orange. (*b*) Longitudinal view of density corresponding to CCDC105 extracted from the DMT map. (*c*) An atomic model of CCDC105 showing its 16 nm periodicity. CCDC105 was identified using an unbiased search with an *AlphaFold*2 library containing 21 615 predicted models of the mouse proteome. CCDC105 has been confirmed at this location in other sperm DMTs using single-particle analysis cryo-EM (Leung *et al.*, 2023 ▸; Zhou *et al.*, 2023 ▸). (*d*) *AlphaFold*2 model of CCDC105.

More recently, advances in protein-structure prediction and automated model building have upended these past workflows to allow the rapid identification of unknown protein densities (Fig. 3 ▸ *b*). Arguably the most transformative advance has been the development of *AlphaFold*2 (Jumper *et al.*, 2021 ▸) and similar neural network-based methods for structure prediction. The ability to rapidly predict protein structures directly from primary sequences now makes it feasible to create or simply download structural libraries of nearly every protein in the proteome of an organism. This expansion of confident protein predictions can be leveraged to identify unknown density in cryo-EM maps. For example, the *BALBES* database can be substituted with an *AlphaFold*2 library in domain-recognition workflows, providing a way to perform domain-based recognition with entire proteomes (Fig. 3 ▸ *b*, left). This approach, using *COLORES* (Wriggers *et al.*, 1999 ▸) as the search program, was recently used to identify CCDC105 and other MIPs in the cryo-ET density of the mouse sperm DMT (Chen, Shiozaki *et al.*, 2023 ▸; Fig. 4 ▸). Importantly, the success of fold recognition is often more dependent on the quality of the starting models than having high-resolution features. *AlphaFold*2 has therefore opened the door to identifying axonemal proteins within lower resolution maps, although without side-chain-level details the risk of misidentification remains, especially among paralogous proteins.

Equally as impactful has been the emergence of *de novo* model-building software such as *DeepTracer* (Pfab *et al.*, 2021 ▸; Chang *et al.*, 2022 ▸) and *ModelAngelo* (Jamali *et al.*, 2024 ▸) that exploit deep-learning methods and can build into any cryo-EM density with resolved side chains, regardless of sequence information (Fig. 3 ▸ *b*, right). One of the advantages of these approaches, as well as model-building aids such as *findMy­Sequence* (Chojnowski *et al.*, 2022 ▸), is that they calculate probabilities for all 20 amino acids for each built residue of every chain. These probability profiles provide better queries for protein identification than single solutions (Mistry *et al.*, 2013 ▸). The power of this approach was illustrated by the identification of four radial spoke proteins (now referred to as RSP24, RSP25, RSP26 and RSP27) and two CA proteins (FAP92 and FAP374) that were unable to be assigned using traditional approaches (Jamali *et al.*, 2024 ▸). The development of *AlphaFold*2 and automated model-building software have not only impacted the field of cilia biology but also the structural biology of large protein assemblies in general.

### 3.3. Model refinement of axonemal complexes

Refining atomic models of axonemal complexes remains a substantial challenge because of their size and the fact that the composite maps exhibit a large range of local resolutions. For the 96 nm repeat of the DMT, its large size prevents refinement of the complex as a single model even after reducing the map dimensions to the smallest cuboid capable of containing the full length of the repeat. Because of these issues, models of 96 nm axonemal assemblies currently need be split into local regions and refined into the cryo-EM density as separate subregions. To allow the refined models to be recombined into a full assembly, these subregions must overlap to avoid the introduction of unrefined interfaces.

## 4. Cryo-ET makes its comeback

Although SPA reconstructions have broken through resolution barriers to describe DMT complexes at sub-4 Å resolution, recent breakthroughs have primed cryo-ET to return as the premier method for imaging native axonemes at high resolution. This section will highlight recent advances in cryo-ET imaging of axonemes and explore its exciting potential.

### 4.1. Using cryo-ET to study axonemes at high resolution

By the mid-2010s, SPA cryo-EM could yield resolutions of ∼3 Å for protein complexes, yet cryo-ET and subtomogram averaging was limited to subnanometre resolution only for large, highly symmetric complexes in thin ice (Kühlbrandt, 2014 ▸). The additional inelastic scattering generated by thicker ice (>100 nm) and the electron damage caused by repeated imaging during tilt-series acquisition precluded high-resolution reconstructions for most cryo-ET samples. A leap towards subnanometre-resolution reconstructions of the axoneme was achieved in 2019 by combining the strengths of cryo-ET with SPA cryo-EM through a process called Tomography-Guided 3D Reconstruction of Subcellular Structures or TYGRESS (Song *et al.*, 2020 ▸). TYGRESS involves acquiring two data sets per target: firstly a single image at 0° tilt with a high dose and secondly a traditional tilt series captured at a lower dose. Following collection, the tilt series is used to create sub­tomogram averages, from which 3D coordinates and alignment angles are extracted. The 3D coordinates are subsequently used to pick particles from the 0° tilt, high-dose image and obtain alignment angles to initialize refinement with SPA methods. Therefore, using this method, the 3D position and initial alignments of each particle are derived from the tilt series, while the higher signal-to-noise ratio 0° initial image is used for high-resolution reconstruction. This general method is particularly well suited to determining structures from highly crowded micrographs. Applied to the *T. thermophila* axoneme, TYGRESS was able to reconstruct the DMT to an average resolution of 12 Å, a significant improvement at the time that clearly resolved individual subcomplexes and some large MIPs (Song *et al.*, 2020 ▸). Although impressive, these reconstructions did not allow accurate atomic model building.

Subsequent improvements in cryo-ET methodology have produced several subnanometre reconstructions of sperm DMTs, including ∼5 Å resolution reconstructions of mouse sperm DMTs (Chen, Shiozaki *et al.*, 2023 ▸; Chen, Greenan *et al.*, 2023 ▸; Tai *et al.*, 2023 ▸; Fig. 4 ▸ *a*). This work has opened the possibility of studying ciliary axonemes by cryo-ET at high resolution. One major advance was the introduction of focused ion-beam (FIB) milling of cryopreserved whole sperm cells to create electron-penetrative lamella. This results in intact axonemes in relatively thin ice with improved signal-to-noise ratios. Axonemes prepared in this way are also typically less ‘flattened’ than when prepared by freezing isolated cilia directly onto cryo-EM grids (Tai *et al.*, 2023 ▸), helping to preserve the geometry of the axoneme and limit damage to the DMTs. Additionally, cryo-ET image-processing workflows have improved by drawing on principles from SPA studies. For studies of DMTs that have resulted in reconstructions below 10 Å, alignments of subtomograms were performed in *RELION*-3 or *RELION*-4, which use a Bayesian statistical approach to align particles based on maximizing the likelihood given the observed data and prior information (Zivanov *et al.*, 2018 ▸, 2022 ▸). Recent versions of *RELION* include cryo-ET-specific tools, such as *CtfRefineTomo* and *TomoFrameAlign*, that promise to improve reconstructions further. Excitingly, this resolution breakthrough promises to improve our understanding of axonemal structures in their native environment.

### 4.2. Cryo-ET and axoneme asymmetry

With the dawn of high-resolution cryo-ET imaging of intact cilia, native features such axoneme asymmetry may be observed. Asymmetry underlies many important features of axonemes in different species, such as the DMT–fibrous sheath connections in mammalian sperm, the DMT–para­flagellar rod connections in trypanosomatid-family parasites, and several noted radial asymmetries in *C. reinhardtii* (Dutcher, 2020 ▸; Bastin *et al.*, 1998 ▸; Baccetti & Afzelius, 1976 ▸). Unfortunately, one of the limitations of SPA cryo-EM is that by deconstructing the axoneme into its constituent filaments, the 9 + 2 organization is destroyed and asymmetrical features of distinct DMT microtubules are averaged and lost. Cryo-ET of native axonemes avoids this issue. One recent example is the cryo-ET study of FIB-milled mouse sperm axonemes, in which separate reconstructions of each DMT revealed that only some have radial spokes associated with a large barrel that may have a role in regulating the sperm-specific asymmetric waveform (Chen, Greenan *et al.*, 2023 ▸). Although axoneme asymmetries have been observed for decades, their molecular basis and function are only now coming into focus through recent improvements in resolution and map quality.

### 4.3. Uncovering transient structures on the axoneme

Cryo-ET has also been central to determining structures of the intraflagellar transport (IFT) A and B cargo-trafficking complexes, which transiently associate with the axoneme. These complexes form long oligomers, termed trains, that assemble at the base of the cilium and traffic cargo to the tip in a kinesin-dependent translocation on DMTs called antero­grade trafficking. Upon reaching the tip, the train reassembles and returns to the base in a dynein-dependent process termed retrograde transport. Tomographic analysis has now observed IFT trains in *C. reinhardtii* assembling at the base of the cilium (van den Hoek *et al.*, 2022 ▸) and during active anterograde transport (Lacey *et al.*, 2023 ▸; Jordan *et al.*, 2018 ▸). Subtomogram averaging applied to the anterograde train achieved a nearly subnanometre resolution, allowing model building (Lacey *et al.*, 2023 ▸) that was supported by contemporary single-particle methods (Meleppattu *et al.*, 2022 ▸; Jiang *et al.*, 2023 ▸; Ma *et al.*, 2023 ▸). This work opens the door to capturing the structures of active processes taking place on the axoneme. Future structures of the retrograde IFT train and trains associated with specific cargos have the potential to offer enormous insight into the process of protein trafficking in cilia.

### 4.4. Cryo-ET and primary cilia

Cryo-ET, and the wider techniques of volume electron microscopy (vEM), also provide suitable methods to image primary cilia. Although it is a convention to describe primary cilia as having a 9 + 0 architecture, tomography on primary cilia isolated from MCDK-II cells (Kiesel *et al.*, 2020 ▸) and serial section-imaging studies of IMCD3 kidney cells (Sun *et al.*, 2019 ▸) and *C. elegans* primary cilia (Doroquez *et al.*, 2014 ▸) have demonstrated that the structures of these axonemes are unconventional, with large radial differences in their organization and DMTs that rapidly taper into singlet microtubules. Future subtomogram averaging of the microtubules of primary cilia should provide new information about their microtubule-associated proteins, about which very little is currently known. Because cells typically have only one primary cilium, fluorescent markers may be useful to help focus imaging on intact FIB-milled primary cilia.

## 5. Conclusions

In the past decade, SPA cryo-EM has invigorated the field of ciliary structural biology by allowing the determination of maps with sufficient resolution to build atomic models that have uncovered the identities, locations and interactions of hundreds of axonemal proteins from an array of species and cell types. This information has provided a foundation to understand the molecular effects of ciliopathies and has revolutionized the field more broadly from working with low-resolution information to specific, residue-level interactions. In the next decade, developments in cryo-ET methods and AI-driven software improvements will address questions of *in situ* axoneme biology. We can look forward to high-resolution structures of axonemes in different functional states, resolving the molecular details of axonemal asymmetry, and observing the relationship between motile and primary cilia and their molecular environment (Müller *et al.*, 2023 ▸; Sheu *et al.*, 2022 ▸).
